# Organic Nanozyme‐Embedded Biotherapeutic Thermosensitive Hydrogel for Extracellular GSH Depletion and Postoperative Tumor Suppression

**DOI:** 10.1002/advs.77309

**Published:** 2026-09-03

**Authors:** Cong Jiang, Zifan Yang, Rong Guo, Meng Suo, Guowei Wang, Dong Chen, Lilin Su, Fuhuan Wang, Shipeng Ning, Kelong Fan, Xuan Wang

**Affiliations:** ^1^ Department of Breast Surgery The Third Affiliated Hospital of Kunming Medical University Yunnan Cancer Hospital Peking University Cancer Hospital Yunnan Kunming Yunnan P. R. China; ^2^ Research Center of Nanomedicine Technology The Second Affiliated Hospital of Guangxi Medical University Nanning Guangxi P. R. China; ^3^ Department of Neurosurgery The Union Hospital of Tongji Medical College Huazhong University of Science and Technology Wuhan Hubei P. R. China; ^4^ Department of Sonography The Third Affiliated Hospital of Kunming Medical University Yunnan Cancer Hospital Peking University Cancer Hospital Yunnan Kunming Yunnan P. R. China; ^5^ General Surgery Department No. 920 Hospital of PLA Joint Logistic Support Force Kunming Yunnan P. R. China; ^6^ CAS Engineering Laboratory For Nanozyme Key Laboratory of Biomacromolecules (CAS) CAS Center for Excellence in Biomacromolecules Institute of Biophysics Chinese Academy of Sciences Beijing P. R. China; ^7^ Nanozyme Laboratory in Zhongyuan Henan Academy of Innovations in Medical Science Zhengzhou Henan P. R. China

**Keywords:** bacteria, extracellular GSH, organic nanozyme, postoperative tumor therapy, thermosensitive hydrogel

## Abstract

Postoperative tumor recurrence remains a major challenge in solid tumor treatment, largely attributed to an immunosuppressive tumor microenvironment and the enrichment of extracellular glutathione (GSH) in the tumor bed, which supports tumor cell survival and proliferation. To address these issues, we designed a multifunctional hydrogel (named SEH) loaded with a novel cyanine nanozyme and *Escherichia coli* (*E. coli*). The cyanine nanozyme integrates efficient photothermal therapy (PTT), photodynamic therapy (PDT), and peroxidase‐like enzyme activity. SEH is a temperature‐sensitive agarose hydrogel. Upon light irradiation, PTT and PDT are simultaneously triggered, inducing hydrogel degradation to release the encapsulated nanozymes and *E. coli*. PTT and PDT jointly kill residual tumor cells and *E. coli*, and the death of the latter leads to the release of pathogen‐associated molecular patterns (PAMPs). The nanozymes combined with PDT cyclically produce more reactive oxygen species to deplete extracellular GSH, which disrupts the redox balance of residual tumor cells. Furthermore, released PAMPs recruit immune cells to the tumor site, thereby activating anti‐tumor immune responses and establishing long‐term immune protection. This study provides a novel and effective strategy for postoperative tumor therapy and recurrence inhibition.

## Graphical abstract

A temperature‐sensitive hydrogel loaded with cyanine nanozymes and *Escherichia coli* is locally injected into postoperative tumor resection beds. Upon light irradiation, synergistic PTT and PDT work with cascaded reactive oxygen species to deplete extracellular GSH, eliminate residual tumor cells, and induce robust antitumor immune responses for effective long‐term inhibition of tumor recurrence.

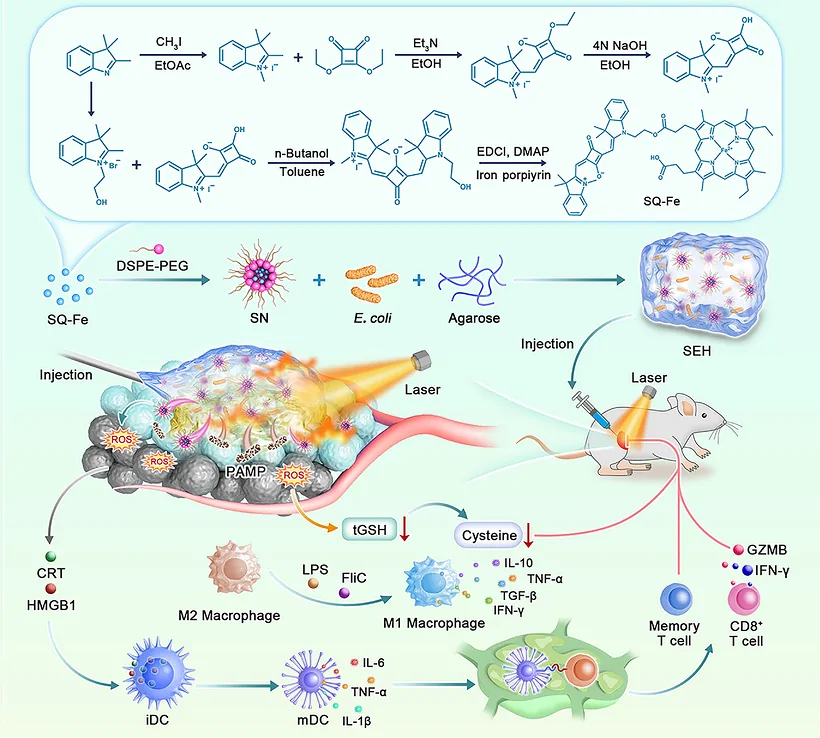

## 1 Introduction

Glutathione (GSH), a tripeptide composed of cysteine, glutamic acid, and glycine, is traditionally recognized as a key intracellular antioxidant, but accumulating evidence has revealed that extracellular GSH in the tumor microenvironment (TME) plays an irreplaceable role in tumor metabolism and recurrence [1, 2, 3, 4]. Unlike the conventional understanding that tumor growth relies on endogenous GSH synthesis, recent studies have demonstrated that extracellular GSH in tumor interstitial fluid is significantly enriched and serves as a crucial cysteine reserve for tumor cells [5]. Through the catalytic degradation of γ‐glutamyl transferase 1 (GGT1), extracellular GSH releases cysteine, which is further integrated into tumor cell protein synthesis and downstream metabolic pathways to support tumor proliferation and survival. Furthermore, extracellular GSH sustains redox homeostasis within the TME. It shields surviving tumor cells from oxidative damage after surgery, increases tumor cell survival, preserves cancer stem cell stemness, and exacerbates local immune suppression [2, 6, 7]. These effects collectively drive tumor relapse. Although surgical resection serves as the primary therapeutic approach for solid tumors, tiny residual tumor lesions combined with the protective, nutritive and immunosuppressive effects mediated by extracellular GSH frequently result in tumor recurrence [4, 8, 9, 10]. Accordingly, targeted elimination of extracellular GSH in postoperative tumor sites can deprive residual tumor cells of energy support, break the intracellular redox equilibrium and reverse immune suppression, which is crucial for inhibiting postoperative tumor recurrence [11, 12].

Nanozymes, as a class of nanomaterials with enzyme‐mimetic activities, have shown great potential in tumor therapy because of their high stability, low toxicity, and tunable functions [13, 14, 15, 16, 17, 18, 19, 20, 21]. Unlike inorganic nanozymes, which are limited by their rigid structure, poor modifiability and unsatisfactory biosafety, organic nanozymes possess distinct merits, including favorable biocompatibility, excellent biodegradability and flexible molecular engineering capability [22, 23]. They more easily achieve multifunctional integration, display lower in vivo toxicity and more desirable metabolic behavior, and can be rationally designed to better match the physiological microenvironment of tumor tissues. Through rational organic engineering, organic nanozymes can be endowed with versatile antitumor functions, achieving synergistic photodynamic therapy (PDT) and enzymatic catalytic therapy. These materials possess distinctive optical responsiveness and enzyme‐mimicking activities and are capable of sustainably consuming intracellular and extracellular GSH via continuous reactive oxygen species (ROS) production. Accordingly, organic nanozymes are well suited for extracellular GSH depletion and postoperative tumor recurrence suppression.

In addition to targeting tumor metabolism, tumor immunotherapy has emerged as another effective strategy to inhibit postoperative recurrence by activating the body's antitumor immune response [24, 25, 26, 27, 28, 29, 30]. Bacteria, especially gram‐negative bacteria such as *Escherichia coli* (*E. coli*), can release pathogen‐associated molecular patterns (PAMPs), including lipopolysaccharides (LPSs), peptidoglycan monomers, and flagellin, upon death [31, 32, 33]. These PAMPs can bind to pattern recognition receptors (PRRs) on immune cells, triggering the synthesis and secretion of proinflammatory cytokines (e.g., TNF‐α and IL‐1), promoting inflammatory programmed cell death (pyroptosis), and further activating innate and adaptive immune responses to eliminate residual tumor cells [34, 35]. However, intravenous injection of bacteria poses significant safety risks, such as systemic infection and inflammatory storms, which limit their clinical application. In contrast, in situ injection of hydrogel‐encapsulated bacteria into the postoperative tumor bed is safer and more effective [36, 37]. Hydrogels with interconnected porous structures can serve as a localized delivery system to achieve sustained release of dead bacteria, avoid systemic dissemination, and effectively recruit immune cells (e.g., dendritic cells and cytotoxic T cells) to the tumor bed, thereby remodeling the immunosuppressive TME and establishing long‐term antitumor immune memory.

**SCHEME 1:**
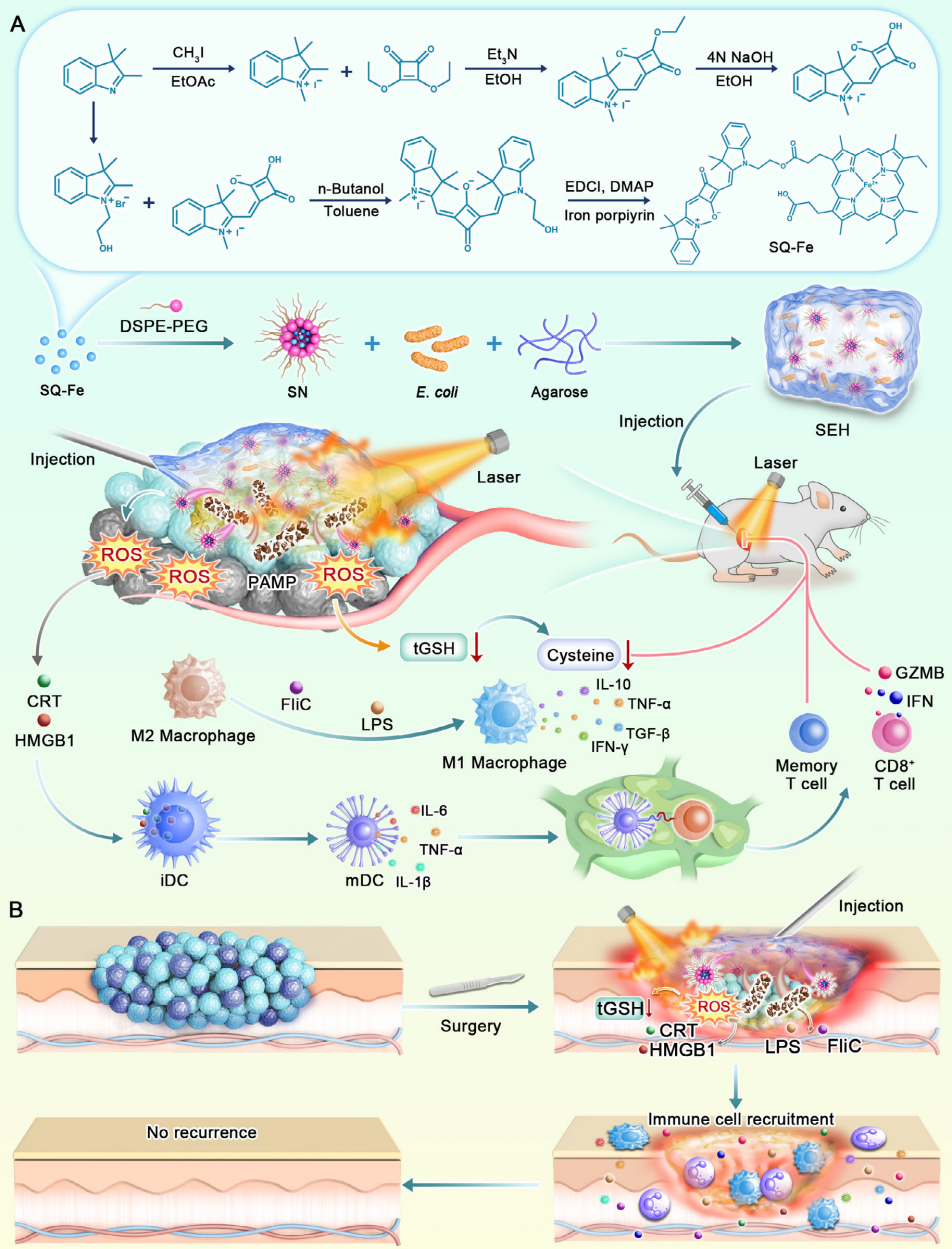
Schematic illustration of organic nanozyme‐embedded biotherapeutic thermosensitive hydrogel for extracellular GSH depletion and postoperative tumor suppression. (A) The synthesis of SQ‐Fe and the construction of the SEH. (B) SEH‐mediated anti‐tumor recurrence.

On the basis of the above considerations, to synergistically address the issues of postoperative tumor recurrence caused by residual tumor cells, an immunosuppressive TME, and insufficient GSH depletion, we designed a hydrogel loaded with organic cyanine nanozymes and *E. coli*, named SEH (Scheme 1). Specifically, the cyanine nanozyme (named SQ‐Fe) used in this study is a novel molecule prepared by our team that can simultaneously achieve efficient photothermal therapy (PTT), PDT, and peroxidase (POD)‐like enzyme activity. SEH is a temperature‐sensitive agarose hydrogel; after in situ injection into the postoperative tumor bed, it undergoes a phase transition to form a stable gel matrix at body temperature, resulting in initial localized retention of the loaded cyanine nanozymes and dead *E. coli*. Subsequent light irradiation not only triggered the photothermal and photodynamic effects of the cyanine nanozymes but also induced the degradation of the temperature‐sensitive agarose hydrogel matrix, thereby triggering the controlled release of the encapsulated cyanine nanozymes and *E. coli*. Moreover, both the photothermal effect and the photodynamic effect generated by light irradiation kill *E. coli*, leading to the simultaneous release of its intracellular contents (including PAMPs and other bioactive substances). Additionally, the photothermal and photodynamic effects together directly kill residual tumor cells through thermal ablation and ROS‐mediated oxidative damage, while the POD‐like enzyme activity of the released cyanine nanozymes, combined with the ROS generated by the photodynamic effect, cyclically produces more ROS to efficiently consume extracellular GSH in the tumor bed, disrupting the redox balance of residual tumor cells and further enhancing their oxidative damage. Moreover, the released contents from dead *E. coli*, together with the degraded hydrogel matrix, synergistically recruit many immune cells, including T lymphocytes, neutrophils, and dendritic cells, to infiltrate the tumor bed. These infiltrated immune cells are activated by PAMPs released from dead *E. coli*, thereby triggering a strong antitumor immune response, establishing long‐term immune protection, and ultimately effectively inhibiting postoperative tumor recurrence. Overall, the SEH integrates multiple therapeutic mechanisms, providing a novel and promising strategy for postoperative tumor treatment.

## 2 Results and Discussion

### 2.1 Preparation and Characterization of SN

**FIGURE 1:**
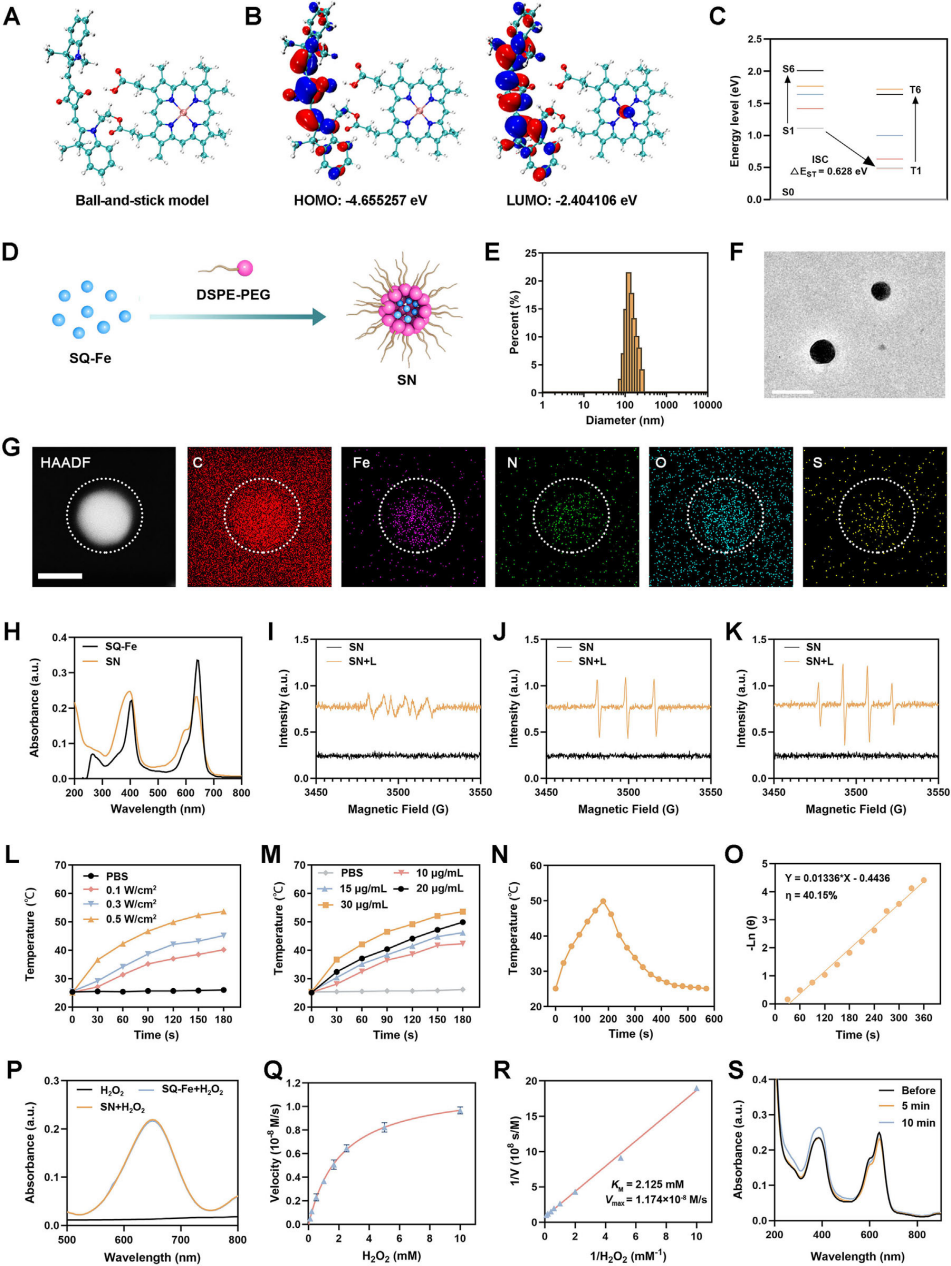
Characterization of cyanine‐based nanozyme (SN) nanoparticles. (A) Ball‐and‐stick molecular model of the cyanine‐based molecule (SQ‐Fe). (B) The highest occupied molecular orbital (HOMO, left) and the lowest unoccupied molecular orbital (LUMO, right) of SQ‐Fe. (C) Energy level diagram of SQ‐Fe showing the singlet‐triplet energy gap. (D) Schematic illustration of the fabrication process of the SN. (E) DLS size distribution of the SN. (F) TEM image of an SN. Scale bar: 200 nm. (G) HAADF‐STEM image and corresponding elemental mapping (C, Fe, N, O, S) of SN. Scale bar: 100 nm. (H) UV–vis absorption spectra of free SQ‐Fe and SN. (I‐K) Electron paramagnetic resonance (EPR) spectra showing ROS generation by SN under different conditions (SN only vs. SN + 660 nm laser irradiation, 0.5 W/cm^2^). (L) Photothermal heating curves of SN solutions at different laser power densities (0, 0.1, 0.3, 0.5 W/cm^2^, 660 nm). (M) Photothermal heating curves of SN solutions at different concentrations (0, 10, 15, 20, and 30 µg/mL) under 660 nm laser irradiation (0.5 W/cm^2^). (N) Photothermal heating and cooling cycle of SN under 660 nm laser irradiation (0.5 W/cm^2^). (O) Linear fitting of the cooling phase to calculate photothermal conversion efficiency (η = 40.15%). (P) POD‐like activity assay of SN using TMB as a substrate in the presence of H_2_O_2_. (Q) Michaelis–Menten kinetic curve of SN‐catalyzed H_2_O_2_ decomposition. (R) Lineweaver–Burk double‐reciprocal plot for determining the kinetic parameters. (S) UV–vis absorption spectra of SN before and after laser irradiation (660 nm laser irradiation, 0.5 W/cm^2^), confirming the retention of characteristic absorption peaks after laser irradiation. The data are shown as the means ± SD (n = 3).

The preparation process of the novel cyanine‐based nanozyme SQ‐Fe is shown in Scheme S1, and we have characterized it, as revealed in Figures S1–S4. The photophysical properties, nanostructure, and therapeutic functions of the SN were systematically characterized, and the key results are shown in Figure 1. First‐principles calculations revealed the molecular structure and electronic distribution of the cyanine molecule (Figure 1A), where the spatial separation of the highest occupied molecular orbital (HOMO; Figure 1B, left) and lowest unoccupied molecular orbital (LUMO; Figure 1B, right), combined with a small singlet‐triplet energy gap (ΔE_ST_ = 0.628 eV; Figure 1C), enabled efficient intersystem crossing (ISC) for ROS generation under 660 nm laser irradiation [38, 39, 40]. SN nanoparticles were successfully prepared via the coassembly of SQ‐Fe and DSPE‐PEG (Figure 1D). Dynamic light scattering (DLS, Figure 1E) and transmission electron microscopy (TEM, Figure 1F) confirmed their uniform spherical morphology, with an average diameter of ∼100 nm. High‐angle annular dark‐field scanning transmission electron microscopy (HAADF‐STEM) and elemental mapping (Figure 1G) verified the homogeneous distribution of C, Fe, N, O, and S, indicating the successful incorporation of both components into the nanocarrier. UV–vis absorption spectra (Figure 1H) revealed that both free SQ‐Fe and the assembled SN exhibited strong near‐infrared (NIR) absorption at ∼660 nm, which matched the irradiation wavelength. Electron paramagnetic resonance (EPR) spectroscopy (Figure 1I–K) confirmed significant ROS generation only in the SN + laser group, verifying light‐dependent photodynamic activity. Under 660 nm laser irradiation (0.5 W/cm^2^), the SN exhibited excellent photothermal performance, as evidenced by rapid temperature elevation with increasing power density (Figure 1L) and concentration (Figure 1M), good photostability during repeated irradiation cycles (Figure 1N), and a high photothermal conversion efficiency (η = 40.15%, Figure 1O). POD‐like activity was validated by TMB oxidation assays (Figure 1P), where SN catalyzed H_2_O_2_ decomposition to generate hydroxyl radicals. Steady‐state kinetic analysis (Figure 1Q,R) revealed a low Michaelis constant (*K*_M_ = 2.125 mM) and high maximum reaction velocity (*V*_max_ = 1.174 × 10^−^^8^ M/s), indicating high catalytic affinity and activity toward H_2_O_2_. After being exposed to light for a long time, the absorption peak of SQ‐Fe did not change significantly, indicating its excellent anti‐quenching property (Figure 1S). Collectively, these results demonstrate that rationally designed SN nanoparticles integrate efficient PTT, PDT, and POD‐like activity, enabling cyclic ROS generation for extracellular GSH depletion, laying a solid foundation for synergistic postoperative tumor therapy and recurrence inhibition.

### 2.2 Preparation and Characterization of SEH

**FIGURE 2:**
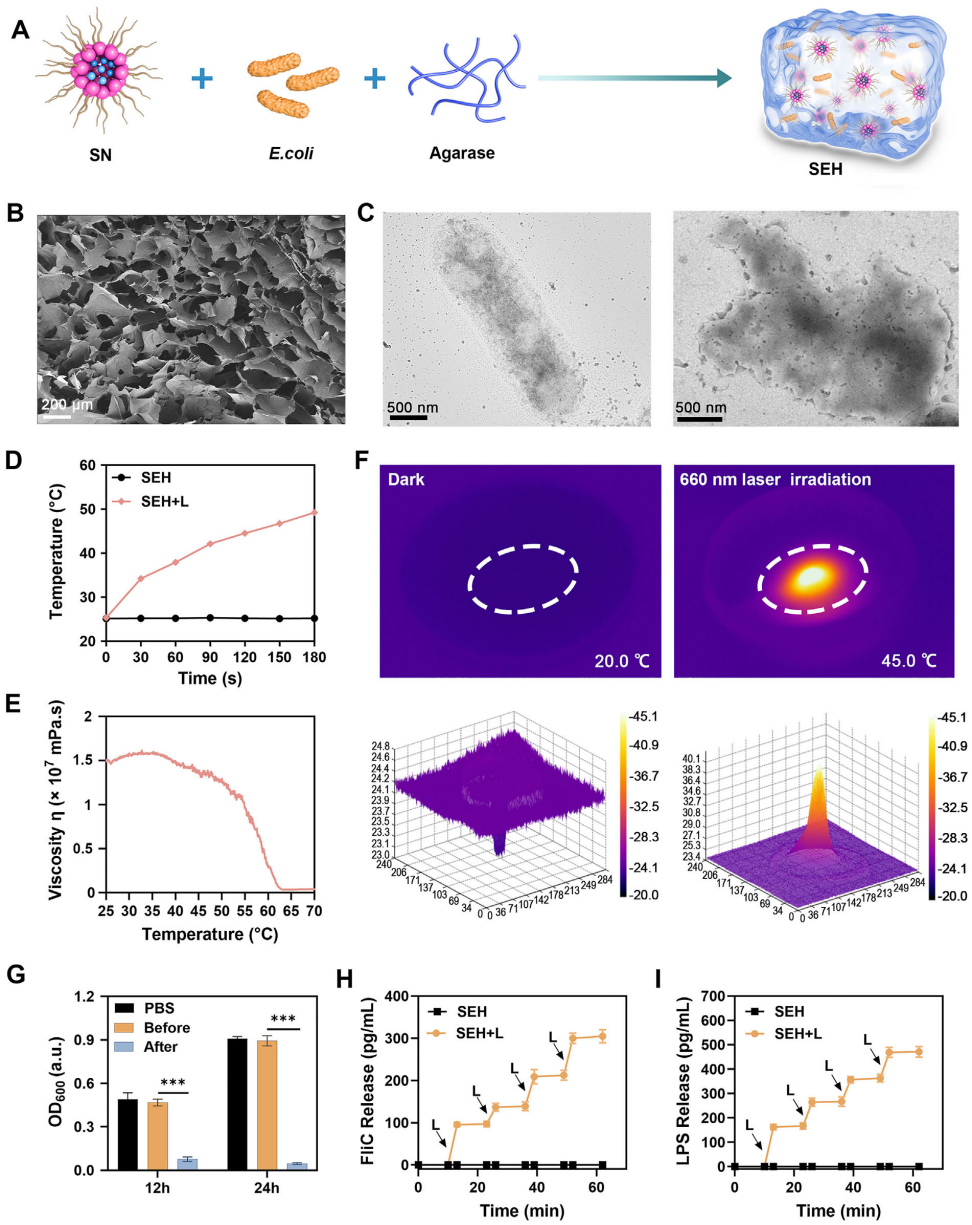
Fabrication and characterization of the SEH. (A) Schematic illustration of the fabrication process of SEH. (B) Scanning electron microscopy (SEM) image showing the porous structure of the SEH. (C) TEM images of *E. coli* before (left) and after (right) light irradiation, showing laser‐induced bacterial damage and lysis. (D) Real‐time temperature monitoring of SEH with or without 660 nm laser irradiation (0.5 W/cm^2^). (E) Viscosity‒temperature curve of the SEH, showing its sol‐gel transition behavior. (F) Infrared thermal images of SEH under dark conditions (left) and after 660 nm laser irradiation (right), showing the increase in photothermal temperature. (G) The bactericidal effect of SEH was evaluated by measuring the OD_600_ of *E. coli* cultures at 12 and 24 h before and after laser treatment. (H) Time‐dependent release profile of LPS from SEH with or without 660 nm laser irradiation. (I) Time‐dependent release profile of flagellin (FliC) from SEH with or without 660 nm laser irradiation. The data are shown as the means ± SD (n = 3). Statistical significance was determined via one‐way ANOVA with Tukey's test: ^***^ *p* < 0.001.

The preparation and characterization of the SEH and its light‐triggered antibacterial and immune‐activating functions were systematically evaluated. First, the fabrication process of the SEH is illustrated in Figure 2A, showing the integration of all the components into a single platform. Scanning electron microscopy (Figure 2B) revealed the porous interconnected structure of the hydrogel, which is favorable for nutrient exchange and the controlled release of loaded agents. TEM (Figure 2C) further confirmed the morphological changes in *E. coli* before and after light irradiation, where laser treatment induced severe structural damage to and lysis of bacterial cells, which is consistent with the photothermal and photodynamic effects of the embedded nanozymes. The photothermal performance of SEH was verified by real‐time temperature monitoring under 660 nm laser irradiation (0.5 W/cm^2^) (Figure 2D). The temperature of the SEH group without a laser was negligible, whereas the temperature of the SEH+L group rapidly increased to ∼50°C within 3 min. The sol‒gel transition behavior of SEH was characterized by viscosity measurements (Figure 2E). The hydrogel remained in a high‐viscosity gel state at physiological temperature, while its viscosity sharply decreased above 60°C, indicating that a light‐induced gel‐sol transition was triggered by photothermal heating. The photothermal effect was further visualized by infrared thermal imaging (Figure 2F), which revealed a maximum temperature of 45.0°C in the irradiated area. The bactericidal effect of SEH was validated by measuring the OD_600_ of *E. coli* cultures (Figure 2G). Both the 12 and 24‐h groups showed significantly reduced bacterial viability after laser treatment, confirming the efficient killing of *E. coli* by synergistic photothermal and photodynamic effects. Furthermore, the release of PAMPs, including LPS and FliC, was monitored over time (Figure 2H,I). The SEH+L group exhibited a gradual and sustained increase in LPS and FliC release upon laser irradiation, whereas the SEH‐only group showed negligible release. These results demonstrate that the SEH hydrogel not only achieves light‐triggered gel degradation and controlled release but also effectively kills loaded *E. coli* and releases immunostimulatory PAMPs, providing a foundation for subsequent in situ immune activation and long‐term tumor recurrence inhibition.

### 2.3 Investigation of the Mechanism of SEH‐Mediated Antitumor Effects In Vitro

**FIGURE 3:**
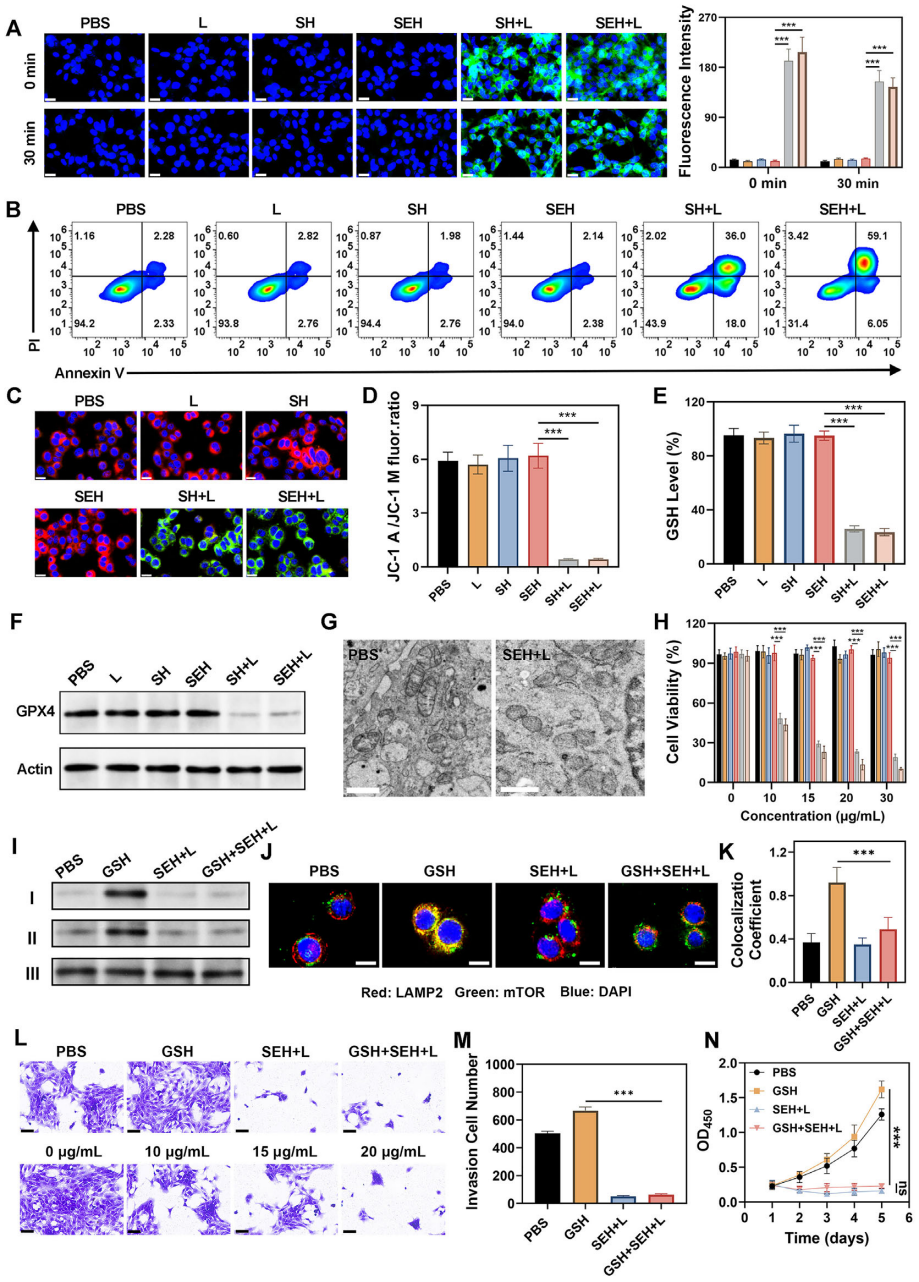
Mechanistic investigation of SEH‐mediated antitumor effects in vitro. (A) ROS generation in 4T1 cells after different treatments (PBS, L, SH, SEH, SH+L, and SEH+L) at 0 and 30 min, as determined by DCFH‐DA staining (green: ROS; blue: DAPI nuclear staining), with corresponding mean fluorescence intensity quantification. Scale bars: 20 µm. (B) Flow cytometry analysis of apoptosis in 4T1 cells after different treatments via Annexin V‐FITC/PI staining. (C) Representative images of 4T1 cell mitochondrial membrane potential detected by JC‐1 staining (red: JC‐1 aggregates; green: JC‐1 monomers) after different treatments. Scale bars: 20 µm. (D) Quantitative analysis of the JC‐1 red/green fluorescence ratio in 4T1 cells after different treatments. (E) Intracellular GSH levels in 4T1 cells after different treatments. (F) Western blot analysis of GPX4 protein expression in 4T1 cells after different treatments, with β‐actin used as the loading control. (G) TEM images showing changes in mitochondrial morphology in 4T1 cells treated with PBS or SEH+L. Scale bars: 1 µm. (H) Viability of 4T1 cells treated with different concentrations (0–30 µg/mL) of various formulations for 24 h. (I) Western blot analysis of p‐4E‐BP1 (I) and p‐S6K1 (II) in 4T1 cells after treatment, with GAPDH (III) as the loading control. (J) Immunofluorescence staining of LAMP2 (red, lysosomal marker), mTOR (green), and DAPI (blue, nucleus) in 4T1 cells after different treatments. (K) Quantitative analysis of the mTOR‐LAMP2 colocalization coefficient in 4T1 cells after different treatments. Scale bars: 10 µm. (L) Representative images of Transwell invasion assays for 4T1 cells after different treatments. Scale bars: 50 µm. (M) Quantitative analysis of invasive cell numbers in 4T1 cells after different treatments. (N) Proliferation curves of 4T1 cells treated with different formulations for 6 days. The data are shown as the means ± SD (n = 3). Statistical significance was determined via one‐way ANOVA with Tukey's test: ^***^ *p* < 0.001.

We first evaluated the in vitro antitumor effects of SEH combined with laser irradiation (SEH+L) in 4T1 cells. DCFH‐DA staining revealed that SEH+L treatment significantly induced intracellular ROS generation, with a marked increase in fluorescence intensity over time (Figure 3A). As ROS are key mediators of both PDT and immunogenic cell death (ICD), this robust ROS burst suggests that SEH+L may act as a potent ICD inducer, which is critical for triggering subsequent antitumor immune responses. Flow cytometry further confirmed that the apoptosis rate in the SEH+L group was significantly greater than that in the other control groups (Figure 3B), indicating that the combination of SEH and laser effectively drives tumor cells to undergo apoptosis. Moreover, JC‐1 staining revealed that SEH+L treatment notably reduced the mitochondrial membrane potential [41, 42], which was accompanied by decreased intracellular GSH levels and downregulated expression of the ferroptosis‐related protein GPX4 (Figure 3C–F). The simultaneous loss of mitochondrial membrane potential, GSH depletion, and GPX4 suppression strongly suggest that ferroptosis can overcome resistance to apoptosis in many cancers and synergize with immunotherapy [43, 44, 45]. TEM also revealed typical ferroptotic morphological changes, such as mitochondrial shrinkage (Figure 3G), providing direct ultrastructural evidence of ferroptosis. Cell viability assays demonstrated that SEH+L exerted concentration‐dependent cytotoxicity against 4T1 cells (Figure 3H), highlighting its therapeutic potency even at low doses. Mechanistic studies revealed that GSH stimulation alone could activate the mTOR pathway, as evidenced by the increased phosphorylation of its downstream effectors p‐4E‐BP1 and p‐S6K1, whereas SEH+L treatment significantly inhibited this activation (Figure 3I–K). These findings indicate that SEH+L suppresses the GSH‐mediated mTOR signaling cascade, which is closely associated with tumor cell invasion and metastasis [46, 47]. Consistently, Transwell assays revealed that SEH+L significantly inhibited cell invasion (Figure 3L–M), and cell proliferation curves confirmed that SEH+L effectively suppressed tumor cell proliferation (Figure 3N). These results indicate that SEH combined with laser irradiation exerts potent in vitro antitumor effects by inducing ROS generation, ferroptosis, and apoptosis and by inhibiting GSH‐induced mTOR pathway activation to block tumor invasion and proliferation, thereby providing a multimodal strategy for breast cancer therapy.

### 2.4 SEH Triggers ICD and Promotes Antitumor Immune Activation In Vitro

**FIGURE 4:**
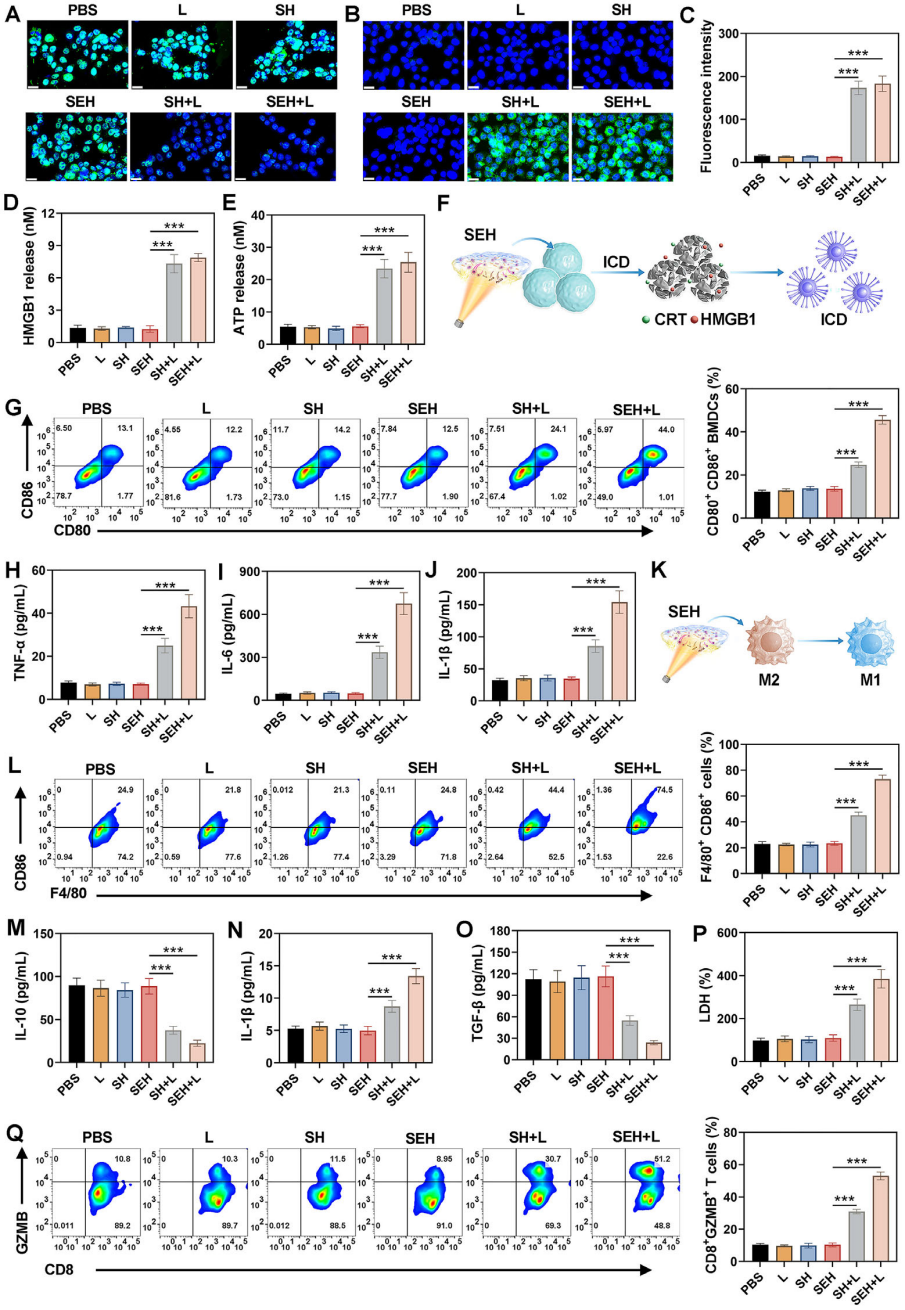
SEH triggers ICD and promotes antitumor immune activation in vitro. (A) Representative immunofluorescence images of HMGB1‐exposed 4T1 cells after different treatments. Scale bars: 20 µm. (B) Representative immunofluorescence images of CRT release in 4T1 cells after different treatments. Scale bars: 20 µm. (C) Quantitative analysis of CRT fluorescence intensity in 4T1 cells after different treatments. (D) Quantitative analysis of HMGB1 release and (E) ATP release from 4T1 cells after different treatments. (F) Schematic illustration of SEH+L‐induced ICD in tumor cells. (G) Flow cytometry analysis of mature CD80^+^CD86^+^ BMDCs after coculture with treated 4T1 cells. (H) ELISA detection of the proinflammatory cytokine TNF‐α in the culture supernatant of treated BMDCs. (I) ELISA detection of the proinflammatory cytokine IL‐6 in the culture supernatant of treated BMDCs. (J) ELISA detection of the proinflammatory cytokine IL‐1β in the culture supernatant of treated BMDCs. (K) Schematic illustration of SEH+L‐induced M2‐to‐M1 macrophage polarization. (L) Flow cytometry analysis of F4/80^+^CD86^+^ M1 macrophages after different treatments. (M) ELISA detection of the anti‐inflammatory cytokine IL‐10 in the culture supernatant of treated macrophages. (N) ELISA detection of the proinflammatory cytokine IL‐1β in the culture supernatant of treated macrophages. (O) ELISA detection of the anti‐inflammatory cytokine TGF‐β in the culture supernatant of treated macrophages. (P) Quantitative analysis of LDH release from 4T1 cells after different treatments. (Q) Flow cytometry analysis of CD8^+^GZMB^+^‐expressing cytotoxic T cells after coculture with mature BMDCs. The data are shown as the means ± SD (n = 3). Statistical significance was determined via one‐way ANOVA with Tukey's test: ^***^ *p* < 0.001.

We next investigated the immunostimulatory effects of SEH+L treatment in vitro. Immunofluorescence imaging (Figure 4A,B) revealed that SEH+L significantly increased the level of calreticulin (CRT) and the release of high‐mobility group box 1 (HMGB1) in 4T1 cells. Quantitative analysis (Figure 4C–E) confirmed that CRT fluorescence intensity was elevated and that the release and secretion of Adenosine Triphosphate (ATP), which are key markers of ICD, were increased. This ICD‐inducing capacity of SEH+L is critical, as CRT exposure acts as an “eat‐me” signal for dendritic cells (DCs) [48, 49, 50, 51], while secreted HMGB1 and ATP serve as “find‐me” signals to recruit and activate antigen‐presenting cells (Figure 4F). Consistently, flow cytometry analysis (Figure 4G) revealed that SEH+L treatment notably promoted the maturation of bone marrow‐derived dendritic cells (BMDCs), as evidenced by the increased proportion of CD80^+^CD86^+^ cells and increased the secretion of proinflammatory cytokines, including TNF‐α, IL‐6, and IL‐1β (Figure 4H–J). Furthermore, SEH+L treatment efficiently induced the polarization of M2‐like macrophages toward the proinflammatory M1 phenotype (Figure 4K), accompanied by an increased proportion of F4/80^+^CD86^+^ M1 macrophages (Figure 4L), reduced secretion of the anti‐inflammatory cytokines IL‐10 and TGF‐β, and increased the production of proinflammatory IL‐1β (Figure 4M–O), which promoted the remodeling of the immunosuppressive TME into an immunostimulatory TME. Moreover, a lactate dehydrogenase (LDH) release assay (Figure 4P) confirmed that SEH+L treatment caused robust tumor cell lysis, which provided sufficient tumor antigens for antigen presentation. Finally, coculture experiments (Figure 4Q) revealed that SEH+L treatment significantly increased the population of CD8^+^GZMB^+^ cytotoxic T cells, indicating the successful priming of tumor‐specific adaptive immunity.

### 2.5 In Vivo Antitumor Efficacy and Immune Activation Induced by SEH+L Treatment

**FIGURE 5:**
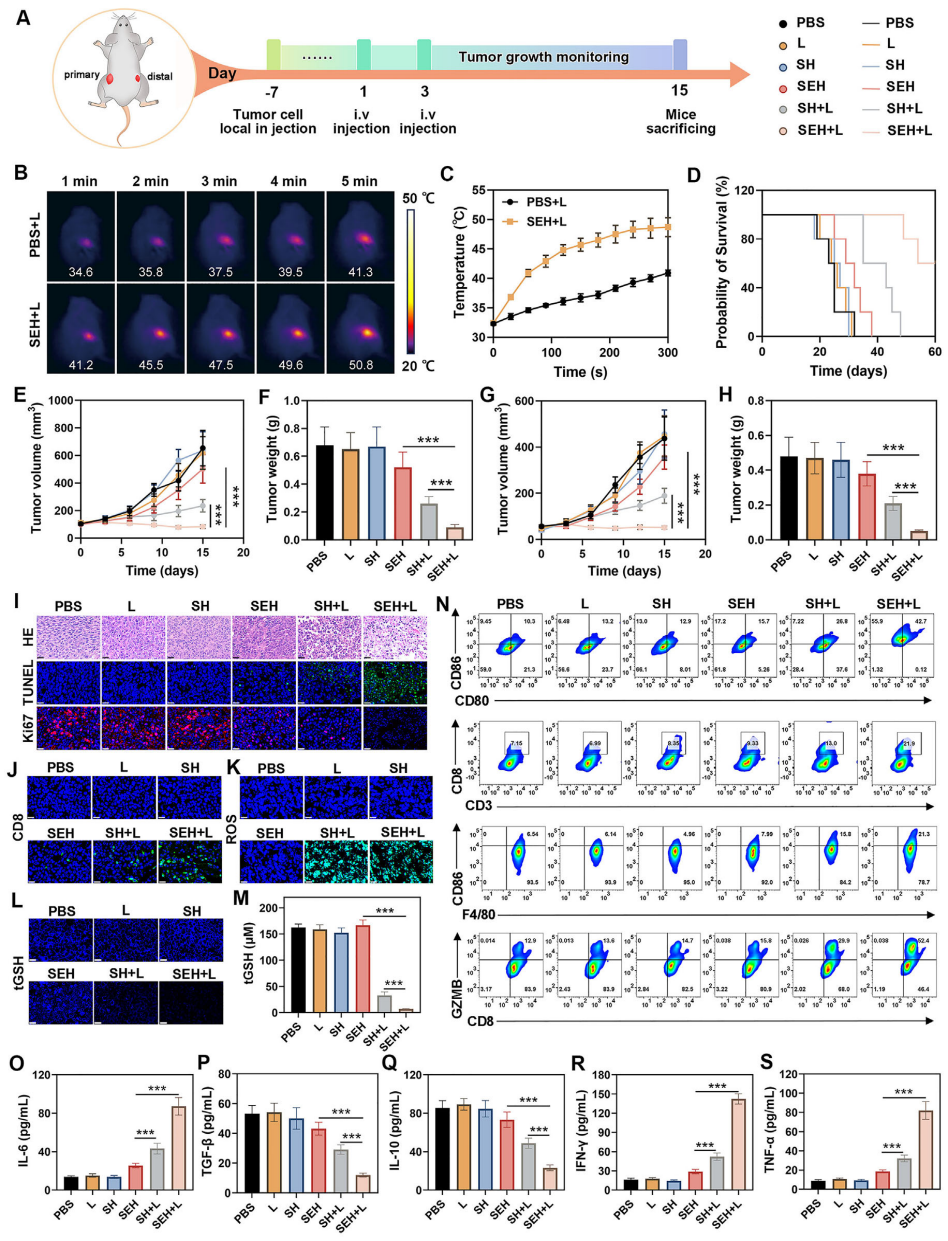
In vivo antitumor efficacy and immune activation induced by SEH+L treatment in 4T1 tumor‐bearing mice. (A) Schematic illustration of the in vivo experimental timeline and treatment schedule for 4T1 tumor‐bearing mice. (B) Infrared thermal images of tumor sites in mice treated with PBS+L or SEH+L under laser irradiation over time. (C) Temperature elevation curves of tumor sites in the PBS+L and SEH+L groups during laser irradiation. (D) Survival curves of 4T1 tumor‐bearing mice following different treatments. (E) Primary tumor growth curves of mice in different treatment groups. (F) Primary tumor weights of the mice at the end of the treatment period. (G) Distal tumor growth curves of mice in different treatment groups. (H) Distal tumor weights of the mice at the end of the treatment period. (I) Representative images of HE, TUNEL, and Ki67 staining of primary tumor tissues from different treatment groups. Scale bars: 20 µm. (J) Representative immunofluorescence images of CD8^+^ T‐cell infiltration in distant tumor tissues. Scale bars: 20 µm. (K) Representative immunofluorescence images of ROS levels in primary tumor tissues. Scale bars: 20 µm. (L) Representative immunofluorescence images of total tGSH levels in primary tumor tissues. Scale bars: 20 µm. (M) Quantitative analysis of tGSH levels in the TIF in different treatment groups. Scale bars: 20 µm. (N) Flow cytometry analysis of immune cell populations, including mature dendritic cells (CD80^+^CD86^+^), infiltrating CD3^+^CD8^+^ T cells, M1 macrophages (F4/80^+^CD86^+^), and cytotoxic CD8^+^GZMB^+^ T cells, in tumor tissues. (O) Serum IL‐6 levels, (P) TGF‐β levels, (Q) IL‐10 levels, (R) IFN‐γ levels and (S) TNF‐α levels in mice after different treatments. The data are shown as the means ± SD (n = 5). Statistical significance was determined via one‐way ANOVA with Tukey's test: ^***^ *p* < 0.001.

We then evaluated the in vivo antitumor efficacy of SEH+L in 4T1 tumor‐bearing mice. An experimental timeline was designed to monitor both primary and distal tumor growth following treatment (Figure 5A). Infrared thermal imaging revealed that SEH+L treatment led to a rapid and significant temperature increase at the tumor site under laser irradiation, confirming its effective photothermal performance (Figure 5B,C). Notably, SEH+L treatment significantly prolonged the survival of tumor‐bearing mice (Figure 5D). Both primary and distal tumor growth were strongly inhibited in the SEH+L group, with significantly reduced tumor volume and weight compared with those in the other control groups (Figure 5E–H). Histological analysis of primary tumors further confirmed the therapeutic effect, as shown by increased numbers of TUNEL‐positive apoptotic cells, decreased numbers of Ki67‐positive proliferative cells, and typical HE staining features of tumor cell necrosis (Figure 5I). Immunofluorescence staining revealed that SEH+L treatment markedly increased CD8^+^ T‐cell infiltration and ROS levels in tumor tissues but significantly reduced total glutathione (tGSH) levels in tumor interstitial fluid (TIF) (Figure 5J–M). A drastic reduction in extracellular tGSH in the TME is particularly critical. Recent studies have demonstrated that extracellular GSH is not merely a bystander antioxidant but also an active regulator of tumor progression and immunosuppression [3, 4, 10]. It directly scavenges ROS generated by therapy, blunts ICD signals, and maintains a redox balance that supports tumor cell survival. More importantly, extracellular GSH has been shown to increase mTOR signaling in tumor cells, promote invasion and metastasis, and impair the function of infiltrating immune cells [52]. By depleting extracellular tGSH in the TIF, SEH+L breaks this key protumorigenic axis, not only by sensitizing tumor cells to phototherapy‐induced oxidative stress but also by removing a critical barrier to effective antitumor immunity. Flow cytometry analysis of tumor‐infiltrating immune cells revealed that SEH+L promoted the maturation of DCs, increased the proportions of infiltrating CD8^+^ T cells and M1 macrophages, and expanded the population of cytotoxic CD8^+^GZMB^+^ T cells (Figure 5N and Figures S5–S8). Moreover, serum cytokine detection demonstrated that SEH+L treatment upregulated the levels of proinflammatory cytokines, including IL‐6, IFN‐γ, and TNF‐α, while downregulating the immunosuppressive cytokines TGF‐β and IL‐10 (Figure 5O–S). Notably, SEH+L maintained a favorable safety profile with no observable weight loss or organ damage (Figures S9–S11). These results collectively demonstrate that SEH+L treatment exerts potent in vivo antitumor effects through effective PTT, ROS generation, and critical extracellular GSH depletion, which together disrupt multiple tumor‐supportive pathways and trigger robust antitumor immune responses to inhibit both primary and distal tumor growth.

### 2.6 SEH Reshapes the Postoperative TME to Drive Robust Antitumor Immunity

**FIGURE 6:**
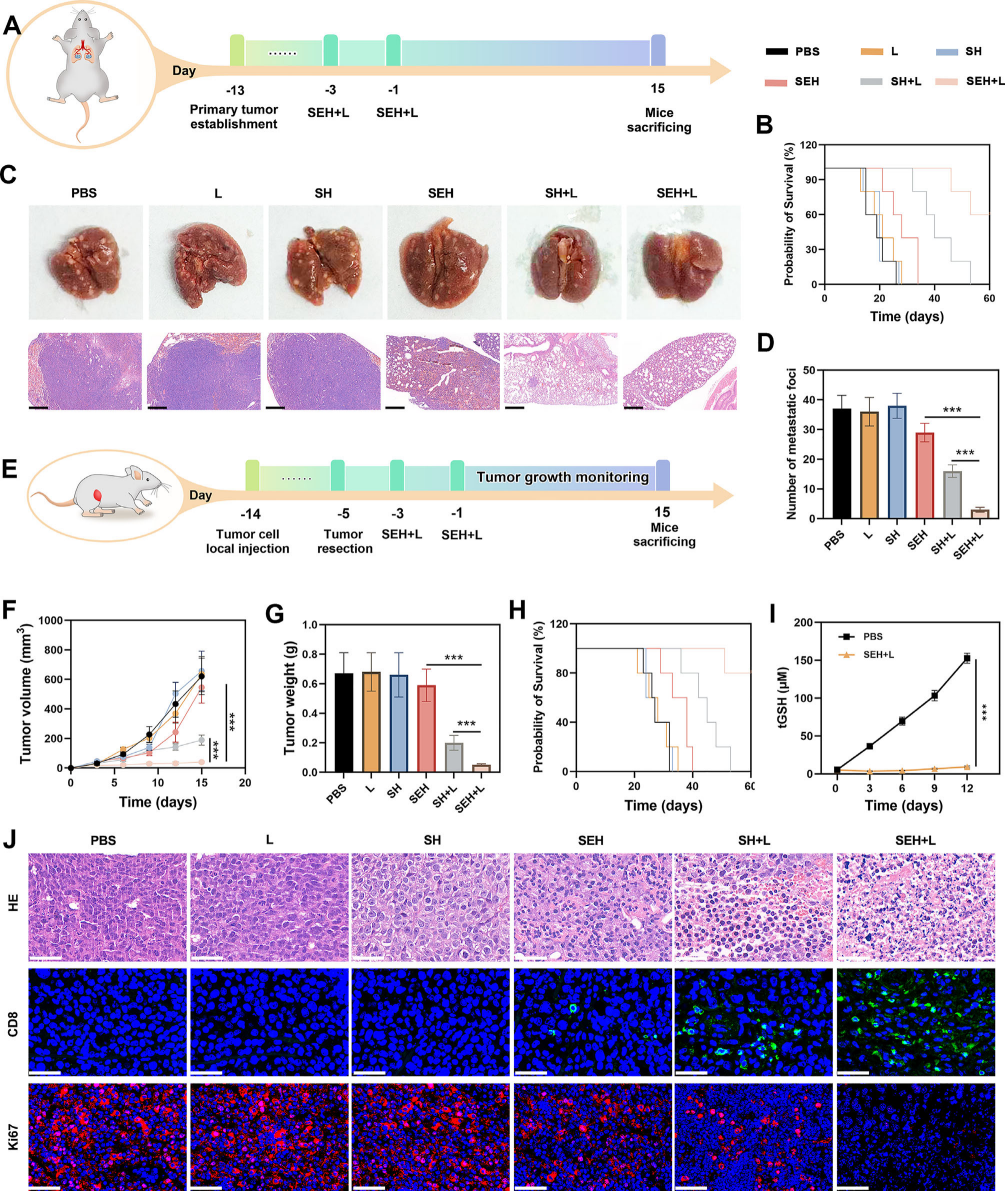
SEH+L inhibits postoperative breast cancer recurrence and distant metastasis in vivo. (A) Schematic timeline of the in vivo experiment for evaluating the antimetastatic efficacy of SEH+L treatment in 4T1 tumor‐bearing mice. (B) Survival curves of antimetastasis model mice following different treatments. (C) Representative images of lung tissues and corresponding HE staining images showing metastatic foci after different treatments. Scale bars: 400 µm. (D) Quantitative analysis of metastatic foci in the lungs of mice in different treatment groups. (E) Schematic timeline of the in vivo experiment for evaluating the antirecurrence efficacy of SEH+L treatment after primary tumor resection. (F) Growth curves of recurrent tumors in different treatment groups. (G) Tumor weights of recurrent tumors at the end of the experiment. (H) Survival curves of mice following different treatments in the postoperative recurrence model. (I) Changes in extracellular tGSH levels over time in the TMEs of the PBS and SEH+L groups. (J) Representative images of HE staining, CD8 immunofluorescence, and Ki67 immunofluorescence in recurrent tumor tissues from different treatment groups. Scale bars: 40 µm. The data are shown as the means ± SD (n = 5). Statistical significance was determined via one‐way ANOVA with Tukey's test: ^***^ *p* < 0.001.

To evaluate the therapeutic potential of SEH+L in preventing both distant metastasis and postoperative recurrence, we first established a 4T1 orthotopic tumor model and treated the mice with SEH+L according to the indicated schedule (Figure 6A). Survival analysis revealed that compared with the other treatments, the SEH+L treatment significantly prolonged the survival of tumor‐bearing mice (Figure 6B). Lung tissue staining and quantitative analysis revealed that SEH+L treatment markedly reduced the number of metastatic foci, demonstrating its potent antimetastatic effect (Figure 6C,D). Next, we assessed the efficacy of SEH+L in preventing postoperative tumor recurrence using a surgical resection model (Figure 6E). Tumor growth curves and endpoint tumor weights revealed that SEH+L treatment strongly inhibited local tumor recurrence after surgery (Figure 6F,G). The survival curves confirmed that the survival time of the mice that received the SEH+L treatment was the longest (Figure 6H). We further measured extracellular tGSH levels in the TIF and found that SEH+L treatment significantly depleted extracellular tGSH, which may disrupt the redox balance that supports residual tumor cell survival (Figure 6I). Histological and immunofluorescence staining of recurrent tumors revealed that SEH+L treatment increased CD8^+^ T‐cell infiltration and decreased the number of Ki67‐positive proliferative cells, indicating enhanced antitumor immunity and suppressed tumor cell proliferation at the surgical site (Figure 6J).

**FIGURE 7:**
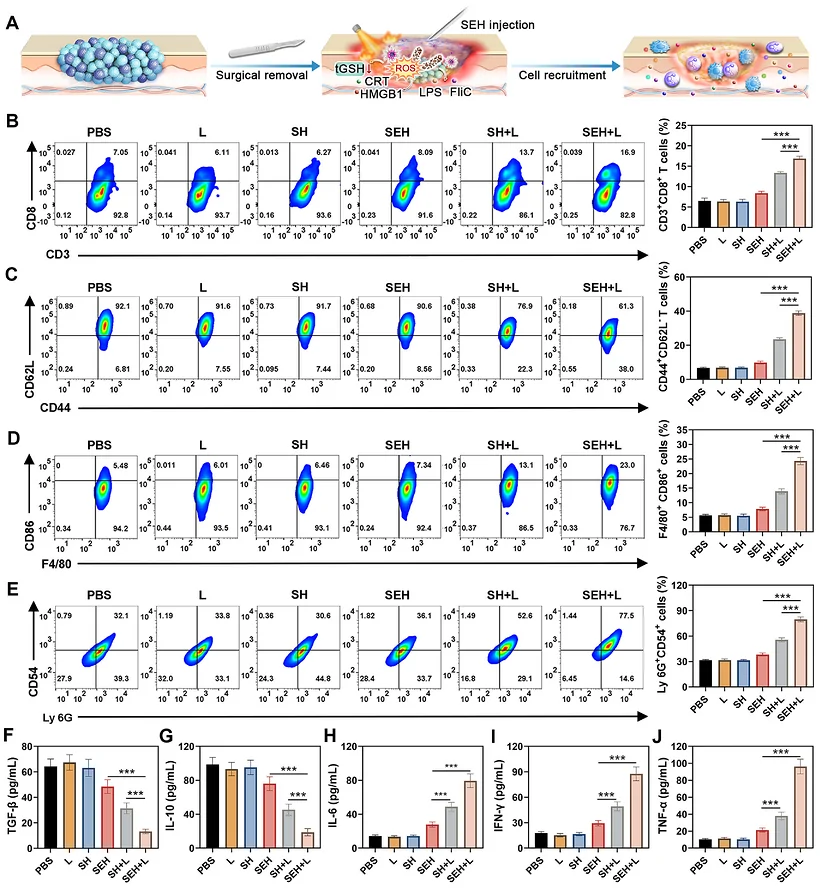
SEH+L remodeled the postoperative TME to promote antitumor immunity. (A) Schematic illustration depicting the mechanism of SEH+L‐mediated modulation of the postsurgical TME, including extracellular tGSH depletion, ICD induction, and immune cell recruitment. (B) Flow cytometry analysis of infiltrating CD3^+^CD8^+^ T cells in the postoperative tumor bed. (C) Flow cytometry analysis of tissue‐resident memory CD62L^−^CD44^+^ T cells in the postoperative tumor bed. (D) Flow cytometry analysis of proinflammatory F4/80^+^CD86^+^ M1 macrophages in the postoperative tumor bed. (E) Flow cytometry analysis of immunosuppressive Ly6G^+^‐positive neutrophils in the postoperative tumor bed. (F‐J) Serum cytokine levels, including those of TGF‐β, IL‐10, IL‐6, IFN‐γ and TNF‐α, in mice from different treatment groups. The data are shown as the means ± SD (n = 5). Statistical significance was determined via one‐way ANOVA with Tukey's test: ^***^ *p* < 0.001.

To further characterize the immunomodulatory effects of SEH+L on the postsurgical tumor site, we investigated the mechanism by which SEH+L modulates the residual TME, including extracellular tGSH depletion, immunogenic cell death induction, and immune cell recruitment (Figure 7A). Flow cytometry analysis of immune cell populations revealed that SEH+L treatment significantly increased the proportions of infiltrating CD3^+^CD8^+^ T cells (Figure 7B) and tissue‐resident memory CD62L^−^CD44^+^ T cells (Figure 7C), indicating the establishment of a robust and persistent antitumor immune response. Additionally, SEH+L treatment promoted the polarization of proinflammatory F4/80^+^CD86^+^ M1 macrophages (Figure 7D) and increased the frequency of CD54^+^Ly6G^+^ N1 neutrophils (Figure 7E), further contributing to an immunostimulatory milieu [53]. Consistently, cytokine profiling revealed that SEH+L treatment significantly decreased the levels of the immunosuppressive cytokines TGF‐β and IL‐10 (Figure 7F,G) but markedly increased the levels of the proinflammatory cytokines IL‐6, IFN‐γ, and TNF‐α (Figure 7H–J).

**FIGURE 8:**
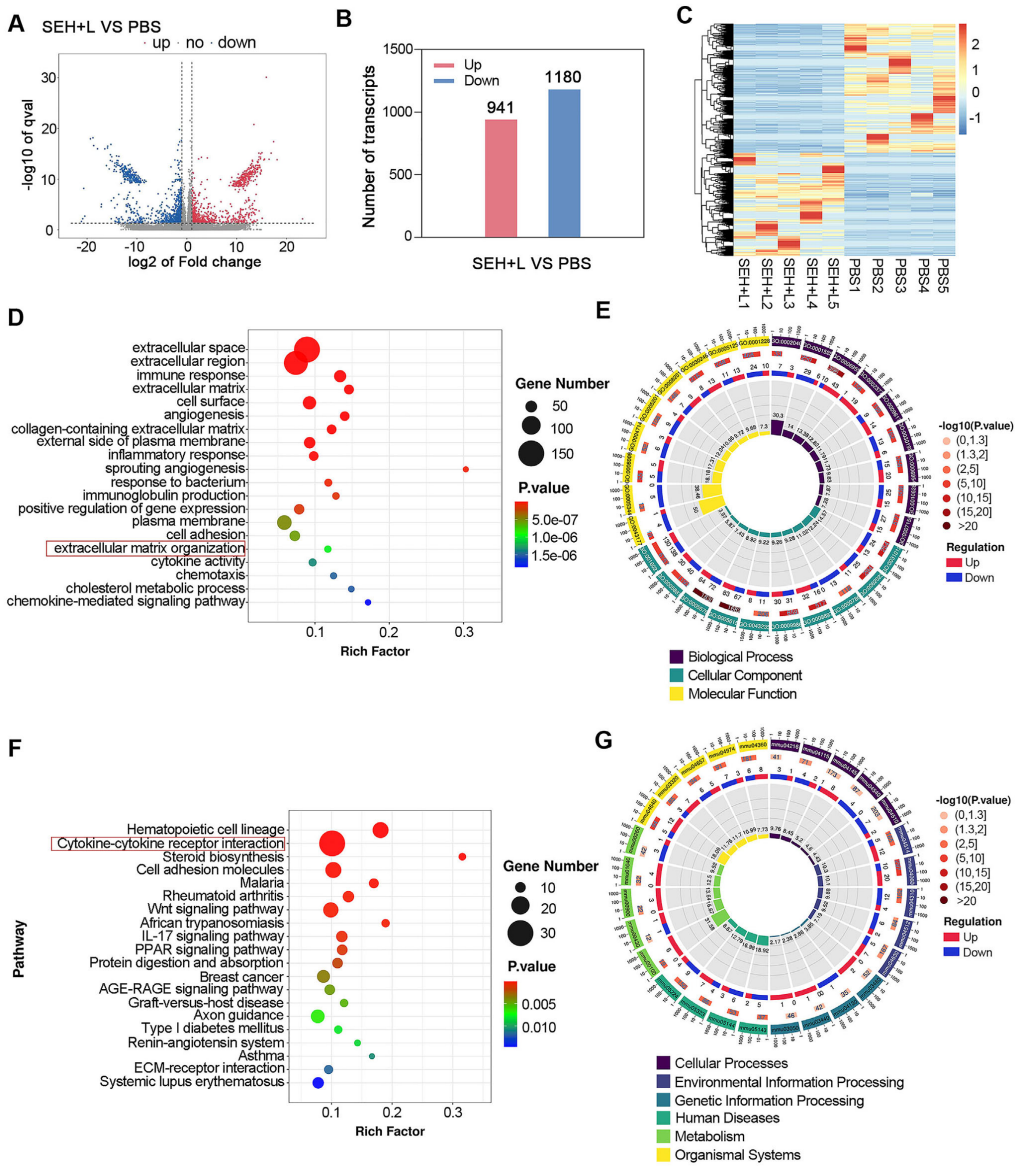
Transcriptomic analysis of recurrent tumors following SEH+L treatment. (A) Volcano plot showing the distribution of differentially expressed transcripts in SEH+L‐treated tumors compared with those in PBS control tumors. (B) Bar graph illustrating the number of upregulated and downregulated transcripts between the SEH+L and PBS groups. (C) Heatmap displaying the hierarchical clustering of differentially expressed transcripts across different treatment groups. (D) GO enrichment bubble plot showing the top enriched biological processes among the differentially expressed genes, with the boxed *extracellular matrix organization* pathway highlighting the remodeling of the TME to support immune cell infiltration. (E) Circular plot summarizing the results of the GO enrichment analysis for the biological process, cellular component, and molecular function categories. (F) KEGG pathway enrichment bubble plot showing the top enriched signaling pathways among the differentially expressed genes, with the boxed *cytokine‐cytokine receptor interaction* pathway representing the core immunomodulatory signaling network driving antitumor immune activation. (G) Circular plot summarizing the results of the KEGG pathway enrichment analysis.

We then performed transcriptomic sequencing to explore the molecular mechanisms underlying the antirecurrence effects of SEH+L treatment following primary tumor resection. Volcano plot analysis (Figure 8A) revealed the distribution of differentially expressed transcripts between the SEH+L and PBS groups, with 941 upregulated and 1180 downregulated transcripts identified (Figure 8B). A hierarchical clustering heatmap (Figure 8C) revealed distinct gene expression patterns between the SEH+L and PBS samples, confirming the consistent transcriptional reprogramming induced by the hydrogel‐based treatment at the postsurgical site. Gene Ontology (GO) enrichment analysis (Figure 8D) revealed that the differentially expressed genes were significantly enriched in biological processes related to the extracellular matrix, immune response, and cell adhesion, which are closely associated with tumor recurrence, local invasion, and immune evasion in the postsurgical niche. The circular GO plot (Figure 8E) further summarizes the enrichment results across biological process, cellular component, and molecular function categories, highlighting the dominant role of extracellular and immune‐related pathways in regulating residual tumor cell survival and recurrence. Kyoto Encyclopedia of Genes and Genomes (KEGG) pathway analysis (Figure 8F) revealed that the top enriched signaling pathways included cytokine‒cytokine receptor interaction, hematopoietic cell lineage, and chemokine signaling pathways, which are key regulators of antitumor immunity and the inflammatory response at the surgical site. Notably, these pathways are critical for remodeling the immunosuppressive postoperative microenvironment, which otherwise supports residual tumor cell outgrowth and recurrence. The circular KEGG plot (Figure 8G) provides a comprehensive overview of these enriched pathways, indicating that SEH+L treatment primarily modulates immune‐related signaling networks in the residual tumor bed. These transcriptomic results demonstrate that SEH+L hydrogel treatment induces profound transcriptional changes in recurrent tumor tissues, particularly by activating immune‐related pathways and modulating extracellular matrix processes at the postsurgical site.

Collectively, these findings demonstrate that SEH+L treatment exerts robust antimetastatic and antirecurrence effects by depleting extracellular tGSH to disrupt the protumorigenic redox balance, triggering potent immunogenic cell death and remodeling the postoperative microenvironment toward an immunostimulatory state. The coordinated activation of DCs, cytotoxic and memory T cells, and proinflammatory macrophages, together with the transcriptomic reprogramming of immune‐related pathways, establishes a durable antitumor immune defense that effectively suppresses residual tumor cell survival, local recurrence, and distant metastasis.

## 3 Conclusions

In conclusion, we have designed a multifunctional SEH that integrates a novel cyanine nanozyme and *E. coli* to achieve synergistic regulation of tumor metabolism, tumor cell killing, and antitumor immunity. The temperature‐sensitive SEH enables in situ retention and controlled release of therapeutic agents after postoperative injection, avoiding systemic side effects associated with intravenous delivery of bacteria. Upon laser irradiation, the cyanine nanozyme‐mediated PTT and PDT synergistically exert multiple therapeutic effects. These processes collectively disrupt the redox balance of residual tumor cells, recruit and activate immune cells, and establish long‐term immune protection, ultimately effectively inhibiting postoperative tumor recurrence. The SEH hydrogel overcomes the limitations of single‐modal therapy and provides a novel, safe, and efficient strategy for postoperative solid tumor treatment.

Future studies will focus on optimizing the composition and properties of SEH hydrogels to further improve their gelation efficiency, drug release kinetics, and biocompatibility. We will also conduct in‐depth in vivo experiments to verify the long‐term effect of SEH and explore its underlying molecular mechanisms. Additionally, efforts will be made to evaluate the biosafety of SEH in large animal models, including the potential risks of bacterial dissemination and inflammatory responses, to lay a solid foundation for its clinical translation.

## 4 Experimental Section

### 4.1 Materials

The DCFH‐DA, Singlet Oxygen Sensor Green (SOSG), DSPE‐PEG2000, Hydroxyphenyl Fluorescein (HPF) and ELISA kits used in this work were purchased from MedChemExpress Co., Ltd. (USA) and Elabscience Biotechnology Co., Ltd. (China). The other solvents used in this work were purchased from Sinopharm Chemical Reagent (China) and Shanghai Macklin Biochemical Technology Co., Ltd. (China).

### 4.2 Animal Ethical Approval Statement

The animal experiments were carried out according to the protocol approved by the Ministry of Health of the People's Republic of PR China and were approved by the Administrative Committee on Animal Research of Guangxi Medical University (2025‐KYL (095)).

### 4.3 Preparation and Characterization of SQ‐Fe‐Loaded Liposomes (SNs)

A mixture of SQ‐Fe (1 mg) and DSPE‐PEG2000 (3 mg) and chloroform (1 mL) was sonicated (12 W output) to obtain a clear solution. The mixture was quickly injected into 9 mL of water, and the resulting mixture was vigorously sonicated for 30 min. The mixture was stirred in a fume hood for 12 h to remove the chloroform. SN suspension was subjected to ultrafiltration (molecular weight cutoff of 100 kDa) at 3000 × g for 10 min. The SQ‐Fe loading capacity was calculated by UV–vis spectroscopy on a Lambda 35 UV‒vis spectrophotometer (PerkinElmer). Loading capacity = M_drug_/M_SN_, where M refers to the mass. The size distribution and zeta potential were measured by DLS. The morphology of the synthesized materials was observed with field‐emission TEM (JEM‐F200).

### 4.4 Statistical Analysis

Data analyses were conducted using GraphPad Prism 5.0 software. For variance analysis, one‐way analysis of variance (ANOVA) with Tukey's post hoc test was used. *p* values of <0.05 were considered significant. *p < 0.05, **p < 0.01, ***p < 0.001.

## Author Contributions

**Dong Chen**: investigation, formal analysis. **Cong Jiang**: investigation, formal analysis, visualization, writing – original draft, writing – review and editing. **Zifan Yang**: investigation, formal analysis, writing – original draft, writing – review and editing. **Rong Guo**: investigation, formal analysis. **Lilin Su**: investigation, formal analysis. **Guowei Wang**: investigation, formal analysis. **Meng Suo**: investigation, formal analysis. **Shipeng Ning**: conceptualization, formal analysis, supervision, funding acquisition, project administration, writing – review and editing. **Fuhuan Wang**: investigation, formal analysis. **Kelong Fan**: conceptualization, formal analysis, supervision, project administration, writing – review and editing. **Xuan Wang**: conceptualization, formal analysis, supervision, funding acquisition, project administration, resources, writing – review and editing.

## Conflicts of Interest

The authors declare no conflicts of interest.

## Supporting information




**Supporting File**: advs77309‐sup‐0001‐SuppMat.docx.

## Data Availability

The data that support the findings of this study are available from the corresponding author upon reasonable request.
