# Supplementary material for: Organic Nanozyme‐Embedded Biotherapeutic Thermosensitive Hydrogel for Extracellular GSH Depletion and Postoperative Tumor Suppression
Source: Adv Sci (Weinh). 2026 Sep 3:e77309. Online ahead of print. doi: 10.1002/advs.77309 (PMC13542391; doi:10.1002/advs.77309)
Supplement: Supplementary file 1 [file ADVS-9999-e77309-s001.docx]

Supporting Information

**Organic nanozyme-embedded biotherapeutic thermosensitive hydrogel for extracellular GSH depletion and postoperative tumor suppression**

*Cong Jiang, Zifan Yang, Rong Guo, Meng Suo, Guowei Wang, Dong Chen, Lilin Su, Fuhuan Wang, Shipeng Ning*, Kelong Fan*, Xuan Wang**

**Experimental Procedures**

**Preparation and characterization of SQ-Fe**

**Instruments**

^1^H and ^13^C nuclear magnetic resonance (NMR) spectra were measured on a Bruker AVANCE 300 M and 500 M spectrometers, respectively, using DMSO-*d_6_* or CDCl_3_ as the solvent, and tetramethylsilane (TMS; *δ* = 0 ppm) was chosen as the internal reference. High-resolution mass spectrometry (HRMS) was performed on a Bruker ultrafleXtreme mass spectrometer system operated in matrix-assisted laser desorption/ionization–time-of-flight (MALDI-TOF) mode. UV-vis spectra and fluorescence spectra were measured with a HORIBA Duetta spectrometer.

**Synthesis of 1,2,3,3-tetramethyl-3H-indol-1-ium (2)**

2,3,3-trimethyl-*3H*-indole (2.0 g, 12.6 mmol) and iodomethane (8.9 g, 63.0 mmol) were added to a pressure-resistant bottle, followed by the addition of 30 mL of ethyl acetate. The mixture was heated to reflux overnight. After the solution was cooled to room temperature, the precipitate was collected by filtration and washed with ethyl acetate to yield brown solid **2** (3.6 g) in 95% yield.

**Synthesis of (E)-2-ethoxy-3-oxo-4-((1,3,3-trimethyl-3H-indol-1-ium-2-yl)methylene)cyclobut-1-en-1-olate (3)**

Compound **2** (3.0 g, 10 mmol) and 3,4-diethoxycyclobut-3-ene-1,2-dione (1.6 g, 9.9 mmol) were dissolved in 25 mL of ethanol, after which triethylamine (7.0 mL, 50 mmol) was added. The reaction mixture was heated to reflux for 2 h, and the solution rapidly turned yellow during the reaction. After cooling, the solvent was evaporated under vacuum. The residue was extracted with ethyl acetate, and the organic layer was thoroughly washed with distilled water and brine. The ethyl acetate layer was collected and concentrated under vacuum. The crude product was purified by flash column chromatography (eluent: 10% ethyl acetate/*n*-hexane). After removal of the solvent, target compound **3** was obtained as a yellow solid (3.3 g) with a yield of 78%.

^1^H NMR (300 MHz, CDCl_3_) *δ* 7.32–7.25 (m, 2H), 7.09 (t, *J* = 7.4 Hz, 1H), 6.92 (d, *J* = 8.0 Hz, 1H), 5.38 (s, 1H), 4.91 (q, *J* = 7.1 Hz, 2H), 3.40 (s, 3H), 1.58 (s, 6H), 1.54 (d, *J* = 7.1 Hz, 3H).

**Synthesis of (E)-2-hydroxy-3-oxo-4-((1,3,3-trimethyl-3H-indol-1-ium-2-yl)methylene)cyclobut-1-en-1-olate (4)**

Compound **3** (3 g, 6.0 mmol) was dissolved in 15 mL of ethanol in a 50 mL round-bottom flask. A 4 N sodium hydroxide solution (5 equivalents) was added, and the mixture was refluxed for 2 h. After the reaction mixture was cooled, the solvent was removed under vacuum. The residue was acidified with diluted hydrochloric acid, extracted with chloroform and then washed sufficiently with brine. Hydrolysis product **4** was obtained as a red solid in 88% yield.

**Synthesis of 1-(2-hydroxyethyl)-2,3,3-trimethyl-3H-indol-1-ium (5)**

2,3,3-trimethyl-*3H*-indole (2.0 g, 12.6 mmol) and 2-bromoethanol (7.8 g, 63.0 mmol) were charged into a pressure-resistant bottle with 30 mL of ethyl acetate. The mixture was heated to reflux overnight. Upon cooling to room temperature, the precipitate was collected by filtration and washed with ethyl acetate to give brown solid **5** (3.3 g) in 92% yield.

**Synthesis of (E)-2-(((E)-1-(2-hydroxyethyl)-3,3-dimethylindolin-2-ylidene)methyl)-3-oxo-4-((1,3,3-trimethyl-3H-indol-1-ium-2-yl)methylene)cyclobut-1-en-1-olate (6)**

Compound **5** (283 mg, 1 mmol) and compound **4** (396 mg, 1 mmol) were dissolved in 30 mL of toluene/*n*-butanol mixed solvent (1:1, *v*/*v*) in a round-bottom flask equipped with a condenser. The reaction mixture was refluxed for 12 h. After complete removal of the solvent, the crude product was purified by flash column chromatography using chloroform/methanol as the eluent to afford bright blue solid **6** (322 mg) with a yield of 55%.

**Synthesis of 2-(2-carboxyethyl)-18-(3-(2-((E)-3,3-dimethyl-2-(((E)-2-oxido-4-oxo-3-((1,3,3-trimethyl-3H-indol-1-ium-2-yl)methylene)cyclobut-1-en-1-yl)methylene)indolin-1-yl)-ethoxy)-3-oxopropyl)-7,12-diethyl-3,8,13,17-tetramethylporphyrin-21,23-diide (SQ-Fe)**

Iron porphyrin (62 mg, 0.1 mmol), compound **6** (58 mg, 0.1 mmol), EDCI (28.7 mg, 0.15 mmol) and DMAP (6 mg, 0.05 mmol) were dissolved in 10 mL of anhydrous DMF. The mixture was stirred at room temperature for 12 h, and the reaction progress was monitored by TLC. After the reaction was completed, the mixture was poured into anhydrous diethyl ether to precipitate a large amount of solid. The solid was collected by vacuum filtration and further purified by silica gel column chromatography (eluent: dichloromethane/methanol = 10:1, *v*/*v*) to yield brown solid **SQ-Fe** (62 mg) with a yield of 58.6%.

^1^H NMR (500 MHz, DMSO-*d_6_*) *δ* 7.50 (s, 4H), 7.30 (s, 6H), 7.15 (s, 4H), 5.88 (s, 2H), 5.74 (s, 2H), 4.16 (s, 4H), 3.76 (s, 4H), 1.69 (m, 25H), 1.24 (s, 2H), 0.85 (s, 2H); ^13^C NMR (126 MHz, DMSO-*d_6_*) *δ* 180.7, 178.5, 169.9, 169.5, 143.8, 142.8, 141.4, 127.9, 127.7, 123.5, 122.0, 110.7, 110.1, 86.5, 86.2, 57.8, 48.7, 48.5, 45.9, 30.5, 26.5; HRMS *m/z*: 1056.4428 [M]^+^ (calcd. for C_63_H_65_FeN_6_O_6_^+^, 1056.4435).

**Scheme S1.** Synthesis route of **SQ-Fe**. Reagents and conditions: (**a**) CH_3_I, EtOAc, reflux, 12 h; (**b**) Et_3_N, EtOH, reflux, 2 h; (**c**) 4 N NaOH, EtOH, reflux, 2 h; (**d**) BrCH_2_CH_2_OH, EtOAc, reflux, 12 h; (**e**) *n*-butanol, toluene, reflux, 12 h; (**f**) iron porphyrin, EDCI, DMAP, DMF, r.t., 12 h.

**Computational details**

The ground-state geometries of the molecules were optimized using density functional theory (DFT). The polarizable continuum model (PCM), with a self-consistent reaction field (SCRF), was used to consider the bulk solvation effects. The time-dependent DFT (TD-DFT) method was utilized at the same level of theory to calculate the energy levels of the singlet and triplet states and their gap (ΔE_ST_) based on the optimized singlet-state geometries. Analytical frequency calculations were also performed at the same level of theory to confirm that the optimized structures were at energy minima. The above quantum chemical calculations were carried out by using the Gaussian 16 program.

**Detection of reactive oxygen species (ROS)**

Electron paramagnetic resonance (EPR) experiments were conducted on a Bruker EMXplus EPR spectrometer. For •O_2_^−^ detection, SN (10 μg) and DMPO (5 mg) were dissolved in 1 mL of methanol. The mixed solution was exposed to a 660 nm laser (0.5 W/cm^2^) for 30 s. The product, DMPO-•O_2_^−,^ was immediately tracked with an EPR spectrometer. For ^1^O_2_ detection, SN (10 μg) and TEMP (2 mg) were dissolved in 1 mL of aqueous solution. The mixed solution was exposed to a 660 nm laser (0.5 W/cm^2^) for 30 s. The product, TEMP-^1^O_2,_ was immediately tracked with an EPR spectrometer. For •OH detection, SN (10 μg) and DMPO (5 mg) were dissolved in 1 mL of aqueous solution. The mixed solution was exposed to a 660 nm laser (0.5 W/cm^2^) for 30 s. The product, DMPO-•OH, was immediately tracked with an EPR spectrometer.

**POD-like activity of SN**

To examine the enzyme-like activity of the SN, catalytic oxidation experiments were performed using a TMB substrate. First, a TMB solution (10 mg/mL) and H_2_O_2_ (10 mM) were prepared in NaAc-HAc buffer at pH 4.0. A solution of 10 μg SN was subsequently added and mixed into the reaction mixture. The absorbance at 652 nm was measured. Kinetic measurements of the nanozymes were conducted following previous reports with modifications. The maximum reaction velocity (*V*_max_) and Michaelis constant (*K*_M_) of the obtained nanozymes were calculated following the Michaelis–Menten equation:

$$V=\frac{V_{\max}*S}{K_{M}+S}$$

*V*_max_ represents the maximal reaction velocity, *S* is the substrate concentration, and *K*_M_ is the Michaelis constant reflecting the affinity of the nanozymes for the substrate.

**Photothermal Conversion Efficiency**

A 660 nm NIR laser (Changchun New Industries Tech. Co., Ltd., Changchun, China) with different irradiation powers was used to stimulate the different concentrations of SN in PBS solution. Photothermal images of the suspensions during laser irradiation were recorded using an infrared thermal imaging system. The laser source was equipped with a 4 mm diameter laser module with adjustable power. The photothermal conversion efficiency (η) was calculated using the following equation^1^:

$$\eta=\frac{hS\left( T_{max}-T_{surr} \right)-Q_{0}}{I(1-{10}^{-A_{\lambda}})}$$

where h is the heat transfer coefficient, S is the surface area of the container, and T_max_ and T_surr_ are the equilibrium temperature and ambient temperature, respectively. Q_0_ is the heat associated with the light absorbance of the solvent, A_λ_ is the absorbance of SN at 660 nm, and I is the laser power density.

**Cancer cell line and bacterial culture**

The 4T1 mouse breast cancer cell line was obtained from the Cell Bank of the Chinese Academy of Sciences and incubated in Dulbecco's modified Eagle's medium (DMEM) supplemented with 10% fetal bovine serum, 100 U/mL penicillin G sodium and 100 μg/mL streptomycin sulfate in a humidified atmosphere containing 5% CO_2_ at 37 °C.

BL21 *Escherichia coli* (*E. coli*) was obtained from our laboratory and was aerobically grown in Luria–Bertani (LB) medium (Servicebio Technology Co., Ltd., China) overnight at 37 °C to reach an OD_600_ ≈ 0.8. Afterward, the bacteria were collected in centrifuge tubes by centrifugation at 5000 rpm for 3 min and were subsequently washed 3 times with 4 mL of PBS. The washed bacteria were resuspended in PBS in 5 mL sterile PE tubes for further use.

**Preparation and characterization of the SN and *E. coli* coloaded hydrogel (SEH)**

The general protocol for hydrogel preparation is as follows. The prepared SN (10 mg of SQ-Fe) and *E. coli* (10^8^ CFU) were added to 10 mL of 2% agarose solution to form SEH. Scanning electron microscopy (SEM) images were captured on a Hitachi FE-SEM S4800 instrument with an acceleration voltage of 3 kV. Rheology experiments were performed on an Anton Paar rheometer. Hydrogel samples at different temperatures were prepared and gently placed in the middle of a 15 mm diameter parallel plate with an appropriate gap. Dynamic oscillatory frequency sweep measurements were conducted at a 1% strain amplitude. To prevent the evaporation of water, a lid was prepared on the top.

**LPS and Flagellin (FliC) release study**

The *in vitro* release profile of LPS and FliC from SEH was determined. Next, 10 mL of SEH containing 10^8^ CFU of *E. coli* was added to the culture dish. To investigate the effects of laser irradiation on release behavior, a release experiment of LPS and FliC was initially performed with or without 0.5 W/cm^2^ 660 nm laser irradiation for 3 min. At the appropriate time points, 100 μL of each sample was collected, and an ELISA kit was used to monitor the released LPS and FliC contents.

***In vitro* antibacterial effect of SEH**

*E. coli* cells were grown to the mid-logarithmic phase in LB medium at 37 °C with shaking and then adjusted to an optical density at 600 nm (OD₆₀₀) of 0.5 in sterile PBS. Bacterial suspensions were incubated with PBS, untreated SN, or SN+ 660 nm laser (0.5W/cm^2^). Afterward, the bacteria were incubated at 37 °C with shaking for 12 h and 24 h. At each time point, the OD₆₀₀ value of each group was measured using a microplate reader, and the bacterial growth curves were plotted.

**Mechanistic investigation of SEH-mediated antitumor effects *in vitro***

4T1 cells were cultured in RPMI-1640 medium supplemented with 10% fetal bovine serum and 1% penicillin‒streptomycin at 37 °C in a 5% CO₂ incubator and then divided into six groups (n = 3) for different treatments: PBS alone, 660 nm laser irradiation alone (0.5 W/cm², 5 min), the SH hydrogel (SQ-Fe concentration 20 μg/mL), the SEH hydrogel (SQ-Fe concentration 20 μg/mL), the SH hydrogel combined with laser irradiation, and the SEH hydrogel combined with laser irradiation. Intracellular ROS levels were detected using a DCFH-DA probe (10 μM) and observed by confocal laser scanning microscopy (CLSM), apoptosis was analyzed by flow cytometry using an Annexin V-FITC/PI staining kit, the mitochondrial membrane potential was evaluated by JC-1 staining, and the ratio of red to green fluorescence was quantified, and the intracellular glutathione (GSH) content was measured using a commercial GSH assay kit at 340 nm with a microplate reader. Total proteins were extracted from treated 4T1 cells using RIPA lysis buffer, quantified by a BCA assay, separated by SDS-PAGE, transferred to PVDF membranes, incubated with primary antibodies against GPX4, p-4E-BP1, p-S6K1, GAPDH and β-actin overnight at 4 °C, incubated with HRP-conjugated secondary antibodies, and the protein bands were visualized by ECL chemiluminescence reagent and analyzed by ImageJ software. Treated 4T1 cells were fixed with 2.5% glutaraldehyde and 1% osmium tetroxide, dehydrated with graded ethanol, embedded in epoxy resin, sectioned into ultrathin slices, and stained with uranyl acetate and lead citrate, and mitochondrial morphological changes were observed by transmission electron microscopy (TEM). The other group of cells was fixed with 4% paraformaldehyde, permeabilized with 0.1% Triton X-100, blocked with 5% BSA, incubated with primary antibodies against LAMP2 and mTOR overnight at 4 °C, incubated with fluorescent secondary antibodies and stained with DAPI, and the colocalization coefficient of mTOR and LAMP2 was analyzed by ImageJ after CLSM imaging.

For the cell viability assay, 4T1 cells were treated with SEH (containing 0, 10, 20, or 30 μg/mL SQ-Fe) for 24 h and incubated with CCK-8 solution for 2 h, after which the absorbance at 450 nm was recorded to calculate the cell viability relative to that of the PBS control group.

For the transwell invasion assay, 1 × 10⁵ treated 4T1 cells were seeded into Matrigel-coated upper chambers with serum-free medium, and complete medium was added to the lower chambers. After 24 h of incubation, the noninvaded cells were removed, and the invaded cells were fixed, stained with crystal violet, counted under a microscope and quantified. A total of 1 × 10⁴ 4T1 cells per well were seeded in 24-well plates and treated with different formulations, and the cell numbers were counted every 2 days for 6 consecutive days to construct cell proliferation curves.

**SEH triggers immunogenic cell death (ICD) and promotes antitumor immune activation *in vitro***

4T1 cells were treated with PBS, 660 nm laser irradiation alone, SH hydrogel, SEH hydrogel, SH + laser and SEH + laser (SQ-Fe concentration 20 μg/mL, laser concentration 0.5 W/cm², 5 min) (n = 3), and calreticulin (CRT) exposure was detected by FITC-conjugated anti-CRT antibody and DAPI staining under CLSM. HMGB1 release was measured by immunofluorescence staining with anti-HMGB1 antibody and quantified by an ELISA kit, and ATP secretion in the culture supernatant was detected using an ATP assay kit. Bone marrow-derived dendritic cells (BMDCs) were isolated from 8-week-old female BALB/c mice, cultured in RPMI-1640 medium supplemented with 20 ng/mL GM-CSF, and then co-cultured with treated 4T1 cells in a Transwell system for 24 h. BMDCs were stained with anti-CD80-BV421 and anti-CD86-APC antibodies and analyzed by flow cytometry to determine the maturation ratio, and the levels of TNF-α, IL-6 and IL-1β in the supernatant were detected by commercial ELISA kits. RAW 264.7 macrophages were treated with the above formulations and stained with anti-F4/80 and anti-CD86 antibodies, and the proportion of M1 macrophages was analyzed by flow cytometry, while the levels of secreted IL-10, TGF-β and IL-1β in the supernatant were measured by ELISA. Murine CD8⁺ T cells isolated from mouse spleens were cocultured with mature BMDCs for 48 h and stained with anti-CD8 and anti-GZMB antibodies, and the proportion of CD8⁺GZMB⁺ cytotoxic T cells was analyzed by flow cytometry. Lactate dehydrogenase (LDH) released from treated 4T1 cells was detected using an LDH assay kit at 490 nm to evaluate the degree of cell lysis.

***In vivo* antitumor efficacy and immune activation induced by SEH plus laser treatment**

Female BALB/c mice aged 4-5 weeks were subcutaneously injected with 5 × 10⁶ 4T1 cells into the right flank to establish primary tumor models and 1 × 10⁶ 4T1 cells into the left flank for distal tumor models. The mice were randomly divided into six groups (n = 5) when the tumor volume reached approximately 100 mm³: PBS, 660 nm laser (L), SH alone, SEH alone, SH + L and SEH + L; all formulations were injected intratumorally, and 660 nm laser irradiation (0.5 W/cm², 5 min) was performed 1 h after injection, with the dosage of SQ-Fe at 20 mg/kg. Tumor temperature during laser irradiation was monitored in real time using an infrared thermal imaging system to record temperature elevation curves. Tumor volume (calculated as length × width²/2) and mouse body weight were measured every 3 days, the mice were sacrificed on day 15, the tumors were dissected and weighed, and mouse survival was recorded during the treatment period. Tumor tissues were fixed with 4% paraformaldehyde, embedded in paraffin, sectioned and stained with hematoxylin‒eosin (HE), TUNEL and Ki-67 for histological analysis, and tumor sections were stained with anti-CD8 antibodies, ROS and monobromobimane for CLSM imaging and quantitative analysis. Tumor tissues were ground into single-cell suspensions and stained with fluorescent antibodies against CD80, CD86, CD3, CD8, F4/80, CD86 and GZMB, and the proportions of mature dendritic cells, CD8⁺ T cells, M1 macrophages and cytotoxic T cells were analyzed by flow cytometry. Mouse serum was collected, and the serum levels of IL-6, TGF-β, IL-10, IFN-γ and TNF-α were measured using commercial ELISA kits following the manufacturer’s protocols.

**SEH reshapes the postoperative tumor microenvironment to drive robust antitumor immunity**

For *the in vivo* antimetastasis assay, 9 × 10⁵ 4T1 cells were injected into the right mammary fat pad of BALB/c mice to establish orthotopic breast tumor models, and the mice were treated with the above formulations when the tumor volume reached approximately 500 mm³. After 15 days, the mice were sacrificed, lung tissues were collected to count metastatic nodules, and lung sections were stained with HE for histological observation.

For the postoperative tumor recurrence model, primary tumors were surgically resected when the tumor volume reached approximately 200 mm³, and the mice were divided into the above six groups. All formulations were injected into the surgical tumor bed, tumor recurrence and tumor volume were monitored, mouse survival was recorded, and extracellular total GSH (tGSH) levels in tumor interstitial fluid (TIF) were measured at different time points. Recurrent tumor tissues were stained with HE, anti-CD8 and anti-Ki67 antibodies for immunofluorescence analysis. Immune cells in the postoperative tumor bed were isolated and stained with antibodies against CD80, CD86, CD8, GZMB, CD62L, CD44, F4/80, CD54 and Ly6G and then analyzed by flow cytometry to evaluate dendritic cell maturation, cytotoxic T-cell activation, memory T-cell formation, M1 macrophage polarization and N1 neutrophil infiltration. Serum levels of TGF-β, IL-10, IL-6, IFN-γ and TNF-α were detected by ELISA to assess systemic immune responses. Total RNA was extracted from recurrent tumor tissues from the PBS and SEH + laser groups (n = 3 per group) for RNA sequencing, after which differentially expressed genes (DEGs) were identified, and Gene Ontology (GO) and Kyoto Encyclopedia of Genes and Genomes (KEGG) enrichment analyses were conducted to explore the biological processes and signaling pathways regulated by SEH + laser treatment.

**References**

(1) Zhu, D.; Lyu, M.; Huang, Q.; Suo, M.; Liu, Y.; Jiang, W.; Duo, Y.; Fan, K. Stellate plasmonic exosomes for penetrative targeting tumor NIR-II thermo-radiotherapy. *ACS applied materials & interfaces* **2020**, *12* (33), 36928-36937. DOI: 10.1021/acsami.0c09969.

**Supplementary figures**

**Figure S1.** ^1^H-NMR spectrum (300 MHz) of compound **3** in CDCl_3_.

**Figure S2.** ^1^H-NMR spectrum (500 MHz) of compound **3** in DMSO-*d6*.

**Figure S3**. **^13^**C NMR spectrum (126 MHz, DMSO-*d6*_,_ 298K) of compound **SQ-Fe.**

**Figure S4**. HRMS of compound **SQ-Fe.**

**Figure S5**. Quantitative flow cytometry analysis of intratumoral DC maturation. The data are displayed as the mean ± SD (n = 5). Statistical significance was determined via one-way ANOVA with Tukey’s test: ****p* < 0.001.

**Figure S6**. Quantitative flow cytometry analysis of CD3^+^CD8^+^ T cells. The data are displayed as the mean ± SD (n = 5). Statistical significance was determined via one-way ANOVA with Tukey’s test: ****p* < 0.001.

**Figure S7**. Quantitative flow cytometry analysis of intratumoral M1-type macrophages. The data are displayed as the mean ± SD (n = 5). Statistical significance was determined via one-way ANOVA with Tukey’s test: ****p* < 0.001.

**Figure S8**. Quantitative flow cytometry analysis of granzyme B (GZMB) expression in CD8+ T cells. The data are displayed as the mean ± SD (n = 5). Statistical significance was determined via one-way ANOVA with Tukey’s test: ****p* < 0.001.

**Figure S9**. Changes in body weight during the treatment period. The data are displayed as the mean ± SD (n = 5).

**Figure S10**. Kidney function markers (A) CRE and (B) BUN and (C) liver function markers (ALT, ALP, and AST) from mice after different treatments. The data are displayed as the mean ± SD (n = 5).

**Figure S11**. Representative HE-stained histological images of the heart, liver, spleen, lung, and kidney from mice treated with PBS or SEH+L. Scale bars: 50 μm.
